# Establishment of a tagged variant of Lgr4 receptor suitable for functional and expression studies in the mouse

**DOI:** 10.1007/s11248-017-0027-0

**Published:** 2017-06-20

**Authors:** Vitezslav Kriz, Michaela Krausova, Petra Buresova, Jan Dobes, Dusan Hrckulak, Olga Babosova, Jiri Svec, Vladimir Korinek

**Affiliations:** Institute of Molecular Genetics, Academy of Sciences of the Czech Republic, Videnska, 1083, 142 20 Prague 4, Czechia

**Keywords:** Genome editing, Hemagglutinin tag, Knock-in, R-spondin, TALENs, Wnt signaling

## Abstract

Leucine-rich repeat-containing G-protein-coupled receptor 4 (LGR4) is produced in a broad spectrum of mouse embryonic and adult tissues and its deficiency results in embryonal or perinatal lethality. The LGR4 function was mainly related to its potentiation of canonical Wnt signaling; however, several recent studies associate LGR4 with additional signaling pathways. To obtain a suitable tool for studying the signaling properties of Lgr4, we generated a tagged variant of the Lgr4 receptor using gene targeting in the mouse oocyte. The modified *Lgr4* allele expresses the Lgr4 protein fused with a triple hemagglutinin (3HA) tag located at the extracellular part of the protein. The allele is fully functional, enabling tracking of Lgr4 expression in the mouse tissues. We also show that via surface labeling, the 3HA tag allows direct isolation and analysis of living Lgr4-positive cells obtained from the small intestinal crypts. Finally, the HA tag-specific antibody can be employed to characterize the biochemical features of Lgr4 and to identify possible biding partners of the protein in cells derived from various mouse tissues.

**Electronic supplementary material:**

The online version of this article (doi:10.1007/s11248-017-0027-0) contains supplementary material, which is available to authorized users.

## Introduction

Leucine-rich repeat-containing G-protein-coupled receptor 4 and related LGR5 and LGR6 proteins represents the B subgroup of transmembrane proteins related to the G-protein-coupled receptors (GPCRs) of the hormone receptor class (Hsu et al. 1998, 2014; McDonald et al. 1998). All LGRs are characterized by a large N-terminal extracellular domain containing tandem arrays of leucine-rich repeat (LRR) units. In contrast to the members of the LGR A and C subgroups that bind hormones to activate heterotrimeric G proteins (e.g., the subgroups are represented by the luteinizing hormone receptor and relaxin hormone receptors, respectively), the LGR4/5/6 receptors interact with secreted Wnt pathway agonists R-spondins (RSPOs) (Carmon et al. 2011; de Lau et al. 2011; Glinka et al. 2011). Wnt signaling potentiation is independent of the G-protein activation or β-arrestin translocation (Carmon et al. 2011; de Lau et al. 2011; Ruffner et al. 2012). Instead, RSPO ligand binding to LGR4/5 leads to cell surface clearance of homologous transmembrane E3 ubiquitin ligases ring finger 43 (RNF43) and zinc and ring finger 3 (ZNRF3). The ligases antagonize both canonical and non-canonical Wnt signaling through ubiquitination and degradation of Wnt receptors (Hao et al. 2012). In the RSPO presence, a specific RSPO–LGR4/5–RNF43/ZNRF3 complex is formed and the RNF43/ZNRF3 activity is suppressed by the complex internalization.

LGR4/5/6 proteins are highly homologous (their sequence identity is more than 50%); nevertheless, their expression pattern and cellular function(s) appear to be only partially overlapping. LGR5 was identified as a gene upregulated by aberrant Wnt signaling in human colon cancer cells (van de Wetering et al. 2002). Subsequent lineage tracing studies performed in genetically modified mice revealed that Lgr5 is specifically produced in epithelial stem cells that are localized at the base of epithelial invaginations (so-called crypts) of the small intestine and colon (Barker et al. 2007). Crypt cells form U-shaped structures comprising several hundred cells. Next to stem cells, each crypt harbors rapidly dividing transit-amplifying cells (progenitors) localized above the stem cell compartment. Additionally, in the small intestine, stem cells are intermixed with the Paneth cells, terminally differentiated cells producing anti-bacterial peptides and enzymes [reviewed in Krausova and Korinek (2014)]. Further studies recognized Lgr5 as a marker of adult stem cells in the stomach, hair follicle, mammary gland, and many other mouse tissues (Barker et al. 2010, 2012; de Visser et al. 2012; Jaks et al. 2008; Plaks et al. 2013; Yee et al. 2013). The other subgroup member, Lgr6, is expressed in the earliest hair placode during mouse embryonic development and in the region interconnecting hair follicles with sebaceous glands in adulthood (Snippert et al. 2010).

In contrast, Lgr4 is broadly expressed in proliferative compartments of many embryonic and adult tissues including the gastrointestinal tract, skin, liver, kidney, pancreas, mammary gland, and bone. Owing to the pleiotropic expression, Lgr4 deficiency is embryonically (or peri/prenatally) lethal (Leighton et al. 2001; Mazerbourg et al. 2004). Besides ubiquitin E3 ligase internalization, there is yet another LGR-related mechanism potentiating Wnt signaling. Upon RSPO–LGR4 interaction, LGR4 binds to the IQ motif containing GTPase activating protein 1 (IQGAP1) and increases IQGAP1 affinity to the cytoplasmic mediator of Wnt signaling Dishevelled (DVL). Subsequent formation of a supramolecular complex involving RSPO–LGR4 and Wnt signalosome “boosts” the canonical Wnt signaling pathway (Carmon et al. 2014). Recently, Luo and colleagues reported that Lgr4 functions as an alternative receptor for RANKL [also known as tumor necrosis factor (TNF) superfamily member 11 (TNFSF11)] (Luo et al. 2016). In addition, bone morphogenetic protein 2 (BMP2) antagonist norrin was identified as a new ligand for LGR4. The effect of norrin-LRP4 coupling on Wnt pathway potentiation is similar as that of the RSPO–LGR4 interaction (Deng et al. 2013). In contrast, Planas-Paz and colleagues have documented that during liver growth and regeneration, RSPO1–Lgr4–Rnf43/Znrf3 signaling does not just augment the Wnt pathway output, but is essential for Wnt signaling (Planas-Paz et al. 2016). Very recently, Yan and co-workers discovered that during intestinal stem cell maintenance and expansion, the Wnt and RSPO ligands play distinct roles (Yan et al. 2017). All the results indicate multiple modes of Lgr4-mediated signaling; however, detailed molecular mechanisms of the signal relay remain to be determined.

In the present study we describe generation of a new mouse strain dedicated to study the Lgr4-dependent function(s). The *Lgr4* locus was modified by homologous recombination in the zygote using the transcription activator-like effector nucleases (TALENs)-based technology and exogenous DNA template. The resulting allele produces the Lgr4 protein fused with a 3HA tag at its N-terminus. The allele is fully functional, enabling easy tracking of Lgr4 expression in adult mouse tissues. Furthermore, since the tag is expressed on the cell surface, it allows direct isolation (and analysis) of living Lgr4-positive cells obtained from the mouse organs. Finally, Lgr4 new biding partners or postranslational modification(s) can be identified by mass spectrometry (MS) analysis of immunoprecipitates obtained using an HA tag-specific antibody.

## Materials and methods

### Animal experiments

Housing of mice and in vivo experiments were performed in compliance with the European Communities Council Directive of 24 November 1986 (86/609/EEC) and national and institutional guidelines. Animal care and experimental procedures were approved by the Animal Care Committee of the Institute of Molecular Genetics (Ref. 63/2013). The genetically modified mice were generated by microinjection of TALEN mRNAs in C57BL/6 J mouse eggs together with the template DNA. The template composed of 738 bp left homology arm, 93 bp sequence encoding the 3HA tag and 691 bp right homology arm was obtained as synthetic DNA (Genescript). TALENs were devised to cleave in the first *Lgr4* exon downstream sequence coding for the signal peptide. TALENs were designed using TAL Effector Nucleotide Targeter 2.0 (https://tale-nt.cac.cornell.edu/) (Cermak et al. 2011; Doyle et al. 2012), assembled using the Golden Gate Cloning system (Cermak et al. 2011), and cloned into the ELD–KKR plasmid as described previously (Flemr et al. 2013). The assembled TALEN constructs were sequenced and transcribed in vitro as described previously (Kasparek et al. 2014). Microinjected eggs were transferred into foster mothers. The presence of the modified *Lgr4* allele was screened from tail biopsies of 3-week-old pups by left and right arm PCR; the PCR products were sequenced. Animals harboring the knock-in allele were crossed with C57BL/6 J wild-type (wt) mice to produce heterozygous and homozygous animals. Lgr5–EGFP–CreERT2 mice [B6.129P2-Lgr5tm1(cre/ERT2)Cle/J] were purchased from The Jackson Laboratory (Bar Harbor, Maine, USA) and genotyped as described in the genotyping protocols of the provider using tail biopsies. Primer sequences are given in “Supplementary Table 1”. The mouse strain carrying the modified *Lgr4* allele will be available via The European Mutant Mouse Archive (EMMA) repository (https://www.infrafrontier.eu/resources-and-services/access-emma-mouse-resources/major-collections).

### Cell transfection and immunocytochemical staining

The 3HA–LGR4–FLAG construct encoding human LGR4 including N-terminal insertion of the 3HA tag (downstream signaling peptide) and with the FLAG tag at its C-terminus was generated in the pK-myc backbone (Valenta et al. 2006) using human *LGR4* cDNA (NM_018490; purchased from OriGene) and a site-directed mutagenesis kit (Stratagene). HeLa cells were seeded in 20% confluency on cover slips in a 24-well dish. The next day, the cells were transfected with the 3HA–LGR4–FLAG construct using Lipofectamine 2000 reagent (Thermo Fisher Scientific). The cells were stained 24 h after transfection. Fixed/permeabilized cell staining: cells were washed with phosphate-buffered saline (PBS), fixed and permeabilized with methanol [20 min, at room temperature (RT)]. Next, the cells were incubated with a mouse anti-FLAG monoclonal antibody (clone M2; Sigma-Aldrich) for 1 h, washed with PBS and incubated with a rabbit anti-HA tag monoclonal antibody (clone C29F4; Cell Signaling Technology) (1 h, RT), washed and incubated with secondary goat anti-mouse antibody (45 min, RT; Alexa Flour 488 dye conjugate; Thermo Fisher Scientific), washed with PBS and incubated with goat anti-rabbit antibody (45 min, RT; Alexa Flour 594 dye conjugate; Thermo Fisher Scientific). Cells were washed with PBS and mounted in mowiol (Sigma-Aldrich). Living cell staining: the primary antibody was added to the culture medium of transfected cells growing on coverslips and incubated for 1 h at 37 °C (5% CO_2_). The dish was washed with PBS and incubated with a rabbit anti-HA monoclonal antibody (1 h, 37 °C, 5% CO_2_), washed with PBS and treated with methanol (20 min, RT). After additional wash, the cells were incubated with the secondary antibodies (45 min, RT), washed in PBS and mounted. The staining was visualized with a Leica DM6000 fluorescent microscope.

### Immunohistochemistry

Organs/embryos were fixed with 4% formaldehyde (Sigma-Aldrich) overnight, dehydrated, and embedded in paraffin. Immunostaining with a primary antibody was performed on 6-µm sections after heat-induced antigen retrieval (steam bath, 20 min, specimens were immersed in Tris-EDTA buffer, pH 9) and blocking endogenous peroxidases and unspecific immunoglobulins. The following primary antibodies were used: anti-HA (rabbit monoclonal, clone C29F4; Cell Signaling Technology), anti-PCNA (rabbit polyclonal; Abcam) anti-GFP (chicken polyclonal; Abcam). Subsequently, the sections were incubated with biotin-conjugated secondary antibodies (Life Technologies) and detected by a peroxidase-based Vectastain ABC kit (Vector) with 3,3′-diaminobenzidine (DAB) substrate (Sigma-Aldrich), counterstained with hematoxylin and mounted in acrylic resin (Sigma-Aldrich).

### Flow cytometry and quantitative RT-PCR (qRT-PCR)

Isolation of the small intestinal and colonic crypts was based on the protocol published by Sato and Clevers (2013). Briefly, small intestine or colon was flashed with PBS and cut longitudinally. The intestinal villi (small intestine) were scratch off using a microscopy slide. The tissue was washed extensively using PBS and incubated with 5 mM EDTA (30 min, 4 °C) to release crypts from the underlying connective tissue. Released crypts were filtrated through a 70-µm strainer (Thermo Fisher Scientific) and spun down. The pellet was processed for immunoblotting/immunoprecipitation or immunostaining. The pellet was incubated in serum-free media with dispase (Corning; 18U, 800RPM, 37 °C, 2 × 10 min). Single-cell suspension was spun down and incubated with an anti-HA biotin-conjugated rabbit monoclonal antibody (biotinylated clone C29F4; 15 min, 4 °C). Subsequently, the antibody was washed with Dulbecco’s Modified Eagle Medium (DMEM; Thermo Fisher Scientific) supplemented with 3% fetal bovine serum (FBS; Sigma-Aldrich) and the cells were incubated (15 min, ice) with streptavidin-allophycocyanine (APC) secondary antibody (BD Biosciences). The antibody was washed and the cells were analyzed by flow cytometry using an Influx high-speed cell sorter (BD Biosciences) and sorted to RNA lysis buffer (Qiagen). Gated areas were evaluated by FlowJo software (Tree Star). RNA was isolated from the sorted cells using an RNAeasy Micro kit according to the manufacturer’s protocol including DNAse treatment (Qiagen) and reverse transcribed using MAXIMA reverse transcriptase (Thermo Fisher Scientific). The LightCycler 480 apparatus and SYBR Green I Master Mix (Roche Applied Science) were employed for qRT-PCR. Primers are listed in Supplementary Table 1.

### Immunoblotting (IB) and immunoprecipitation (IP)

Isolated cryptic cells or entire stomach tissue were homogenized in lysis buffer (50 mM Tris pH 7.4, 150 mM NaCl, 1 mM EDTA, 0.5% NP40) supplemented with protease inhibitor cocktail (Roche). Samples were spun down at 20,000×*g* (10 min, 4 °C). Supernatant was directly mixed with Laemmli sample buffer. For IP, the supernatant was incubated with prewashed anti-HA tag magnetic beads (clone 2-2.2.14; Thermo Fisher scientific) in a carousel (1 h, 4 °C). Subsequently, the magnetic beads were washed three times with complete lysis buffer and twice with lysis buffer without detergent. To avoid centrifugation during the washing steps, magnetic beads were separated by a magnet and gently re-suspended in new buffer by inverting the tube. After the last washing step, the magnetic beads were frozen as a pellet in −80 °C and used for MS analysis. Alternatively, the magnetic beads were re-suspended in 50 µl lysis buffer, mixed with Laemmli sample buffer and used as for IB. Samples were boiled for 10 min and separated by vertical electrophoresis in 10% denaturation acrylamide gel, semidry blotted and incubated with a rabbit anti-HA monoclonal antibody (clone C29F4; Cell Signaling) or rabbit anti-β-actin (whole rabbit serum; Sigma-Aldrich).

### MS analysis

The samples were cleaved directly on beads with 1 µg of trypsin (Sigma-Aldrich) at 37 °C overnight (Masuda et al. 2008). Neutral lost scan mass spectrometry (NLS-MS) analysis was performed according to Hebert et al. (2014). All data were analyzed and quantified with MaxQuant software (version 1.5.3.8) (Cox et al. 2014). The false discovery rate (FDR) was set to 1% for both proteins and peptides, and we specified a minimum length of seven amino acids. The Andromeda search engine was used for the MS/MS spectra search against the *Mus musculus* database (downloaded from Uniprot in March 2015, containing 44,654 entries). Enzyme specificity was set as C-terminal to Arg and Lys, also allowing cleavage at proline bonds and a maximum of two missed cleavages. Dithiomethylation of cysteine was selected as fixed modification and N-terminal protein acetylation and methionine oxidation as variable modifications. The “match between runs” feature of MaxQuant was used to transfer identifications to other LC–MS/MS runs based on their masses and retention time (maximum deviation 0.7 min) and this was also used in quantification experiments. Quantifications were performed with the label-free algorithms. Data analysis was performed using Perseus 1.5.2.4 software (Tyanova et al. 2016).

## Results and discussion

### Generation of Lgr4^3HA/3HA^ mice

**Fig. 1:**
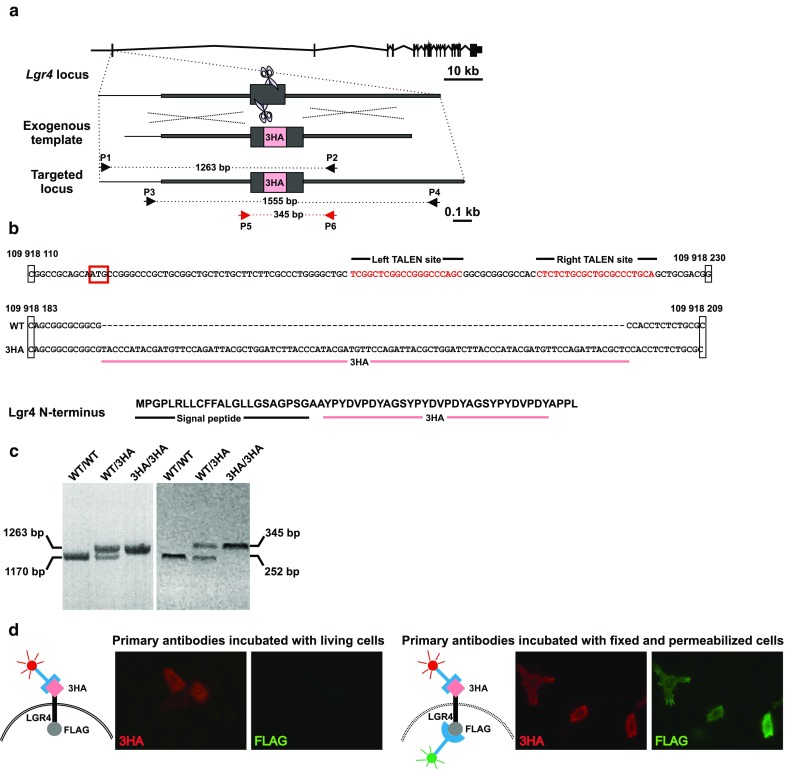
Generation of mice harboring a modified *Lgr4* allele producing the 3HA–Lgr4 fusion protein. **a** The *top* diagram shows TALEN-based genome editing of the *Lgr4* locus; exons are depicted by *black boxes*. A pair of TALENs (scissors pictogram) recognizes and cleaves a specific sequence in the first exon of the gene. The affected locus is repaired by homologous recombination using an exogenous template carrying a portion of the *Lgr4* gene including the 3HA tag sequence (*red box*). Correct targeting (at both ends of the template) is verified by sequencing of PCR products amplified from genomic DNA using two primer pairs: P1 and P2, P3 and P4; primer positions are depicted by *black arrows*. Notice that one primer from each set, i.e., P1 and P4, primes in a sequence that is not present in the targeting construct. For regular genotyping, i.e., upon establishment of Lgr4^+/3HA^ and Lgr4^3HA/3HA^ strains, PCR with P5 and P6 primers (*red arrows*) generating shorter DNA fragments were utilized. **b** Top, TALEN recognition sites (sequence in *red*) in the first *Lgr4* exon; translational start is *boxed* in *red*. The numbers above the sequence indicate nucleotide positions in the mouse genome assembly GRCm38:CM000995.2 (nucleotide numbering in other schemes was also taken from the same genome assembly). *Middle* the nucleotide sequence of the corresponding part of the *Lgr4* locus upon insertion of the 3HA tag encoding sequence. *Bottom* the amino acid sequence of Lgr4 N-terminus fused to 3HA tag. **c** Agarose gel electrophoresis discriminating Lgr4^+/+^ (WT/WT), Lgr4^+/3HA^ (WT/3HA), and Lgr4^3HA/3HA^ (3HA/3HA) animals using P1 and P2 (*left* gel), and P5 and P6 (*right* gel) primers. The gel containing DNA fragments obtained by PCR with primers P3 and P4 that are hardly distinguishable by standard electrophoresis (fragment sizes 1462 bp for wt and 1555 bp for the targeted allele) is not shown. **d** Cell-based functionality test of the tagged LGR4 receptor. Immunofluorescent microscopy images of HeLa cells transfected with the construct expressing human LGR4 protein tagged with the 3HA and FLAG tag at its N- and C-terminus, respectively. A rabbit monoclonal antibody recognizing the 3HA tag or mouse anti-FLAG antibodies were incubated either with living (*left* image) or fixed (and permeabilized) cells (*right* image) grown on coverslips. The retained immunoglobulins were visualized by fluorescently labeled goat anti-rabbit (*red* fluorescence) or mouse anti-FLAG tag (*green* fluorescence) secondary antibodies. Notice that whereas both antibodies stained fixed cells, living cells were recognized only with the 3HA-specific antibody reacting with the extracellular portion of 3HA–LGR4–FLAG fusion protein (see also the schemes on the* left* sides of the images). (Color figure online)

To introduce a single-copy construct into the *Lgr4* locus, we generated a TALEN pair specific for the sequence in the first *Lgr4* exon. Furthermore, we generated a construct containing the 3HA tag sequence flanked by left and right homology “arms” derived from the corresponding regions of the *Lgr4* locus (Fig. 1a). The construct was co-injected with TALEN mRNAs into fertilized eggs. From 373 transferred zygotes, 25 pups were born; sequencing of DNA fragments generated by PCR using genomic DNA isolated from tail biopsies revealed that 11 founders carried the 3HA tag sequence correctly inserted into the targeted locus. The PCR reactions were performed with two primer pairs priming upstream and inside (primers P1 and P2) or inside and downstream (primers P3 and P4) in the genomic region used for the production of the targeting construct. The 3HA tag was placed immediately downstream of the *Lgr4* sequence encoding the signal peptide of the protein (Fig. 1b). To eliminate the possibility of random integration of the construct into the genome, we crossed three mice (two females and one male) with wild-type (wt) C57BL/6 J mice for five generations. For routine PCR-based genotyping, additional primer pairs (P4 and P6) generating a shorter PCR product were utilized (Fig. 1c). In the offspring of all three founders, the *3HA–Lgr4* allele was inherited in a standard Mendelian ratio, implicating a single integration site of the construct. In parallel, we tested the membrane localization and accessibility of the 3HA tag in the context of 3HA–Lgr4 fusion protein. HeLa cells were transiently transfected on coverslips with a construct encoding human 3HA–LGR4 containing a FLAG tag at its C-terminus. To capture the extracellular portion of the receptor, transfected growing cells were briefly incubated with an anti-HA and anti-FLAG antibody. The cells were washed, fixed (and permeabilized), and retained anti-HA or anti-FLAG immunoglobulins were directly stained with differentially labeled secondary antibodies reacting with rabbit (for HA detection) or mouse (FLAG detection) immunoglobulins. In parallel, the transfected cells were first fixed/permeabilized (to make both ends of the protein accessible) and then incubated with the primary and secondary antibodies. The assay confirmed that in living cells, the 3HA epitope was localized extracellularly and the FLAG tag was inaccessible. In contrast, in cells permeabilized prior to staining, both tags were detected (Fig. 1d). The functionality of the 3HA–Lgr4 protein was further confirmed in vivo when heterozygous mice of each founder were intercrossed. Viable and fertile Lgr4^3HA/3HA^ homozygotes for all three original founders were obtained and, subsequently, corresponding homozygous strains were established.

### Immunohistochemical detection of 3HA–Lgr4 in the gastrointestinal tract and skin

**Fig. 2:**
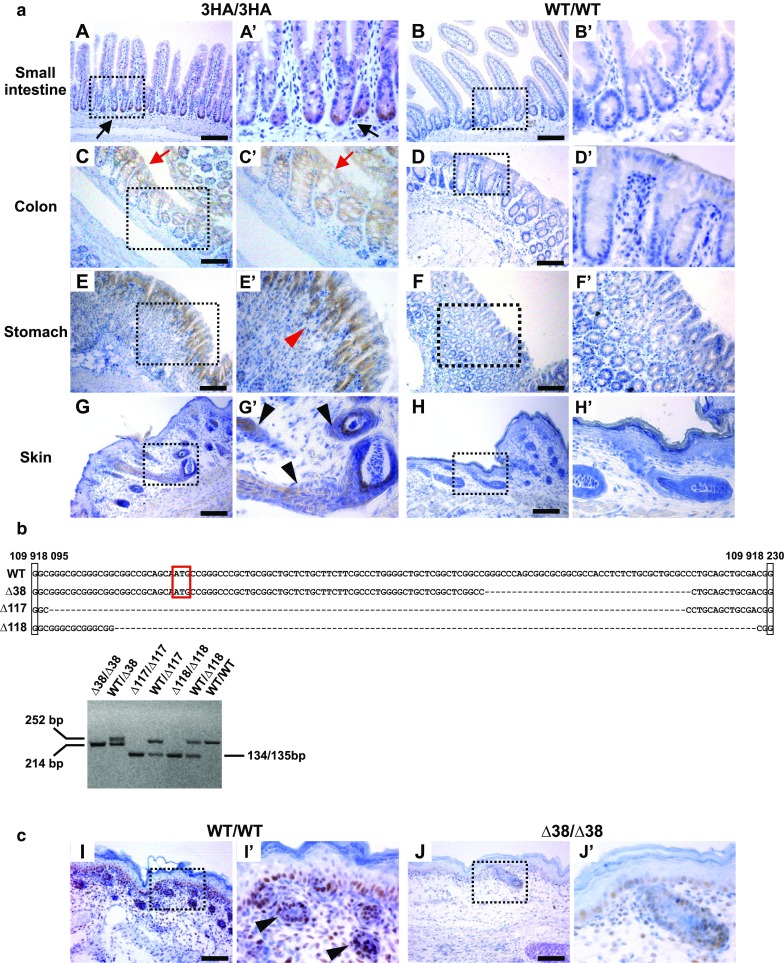
Immunohistochemical analysis of Lgr4^3HA/3HA^ and Lgr4^∆38/∆38^ mice. **a** Paraffin sections of Lgr4^3HA/3HA^ (3HA/3HA) and control Lgr4^+/+^ (WT/WT) animals stained with an HA-tag monoclonal and DAB staining (*brownish* precipitate); specimens were counterstained with hematoxylin (*blue* nuclei). In the small intestine, the 3HA tag was mainly detected at the base of the crypts (*black arrows* in **A**, **A′**), whereas 3HA–Lgr4 production in the colon increased along the crypt axis and was highest on the colonic surface (*red arrows* in **C**, **C′**). In the fundus of the stomach, the staining is mainly localized to the isthmic region (*red arrowhead* in **E′**). In the skin, the positivity is visible in the inner sheath of the hair follicle (*black arrowhead* in **E′**). The specimens were obtained from adult (small intestine, colon, stomach) or new-born (skin) mice. The staining in all three established Lgr4^3HA/3HA^ strains were virtually identical, representative images are shown. **b** *Top* nucleotide sequence corresponding to the wt or ∆38, ∆117, ∆118 mutant alleles; the translational start is *boxed* in *red*. *Bottom* PCR-based genotyping of the Lgr4-deficient mouse strains. Primers P5 and P6 were employed for the analysis. **c** Reduced number of hair follicles (*black arrowhead* in I’) and PCNA-positive proliferating cells (*brown* cell nuclei) in the skin of wt and Lgr4^∆38/∆38^ mouse embryos collected at E18.5. *Scale bar* 100 μm (**A**, **B**, **C**, **D**, **E**, **F**, **G**, **H**, **I**, **J**). The *insets* are enlarged in the corresponding right images (**A′**, **B′**, **C′**, **D′**, **E′**, **F′**, **G′**, **H′**, **I′**, **J′**). (Color figure online)

Our next goal was to test whether the 3HA tag might be visualized by immunohistochemistry. Lgr4 is broadly expressed in various organs including the kidney, stomach, intestine, bladder, heart, brain, bone, male reproductive tract, eye, and skin (Kato et al. 2007; Mazerbourg et al. 2004; Mendive et al. 2006; Mohri et al. 2008). Since the main interest of our laboratory is the digestive tract, we primarily focused on 3HA tag expression in the small intestine, colon, and stomach. In the small intestine, Lgr4 production was tracked by various methods including mRNA in situ hybridization (Mustata et al. 2011), immunohistochemistry (Yi et al. 2013), and by the β-galactosidase (LacZ) reporter expressed from the *Lgr4* locus (de Lau et al. 2011; Mustata et al. 2011). In concordance with the data published previously, the 3HA antigen was detected in all anatomical parts of the small intestine (duodenum, jejunum, and ileum). As expected, the strongest signal was observed in the lower portions of the crypts and the signal intensity decreased along the crypt-villus axis (Fig. 2a; A, A′, B, B′). The expression pattern of 3HA–Lgr4 in the colon showed the opposite gradient, with the strongest signal in differentiated cells located at the apical parts of the crypts and on the colonic surface (Fig. 2a; C, C′, D, D′). The staining pattern was in stark contrast to previously published results based on the Lgr4–LacZ reporter activity (Liu et al. 2013) or mRNA detection (Mustata et al. 2011). Nevertheless, our results matched the staining pattern obtained using Lgr4-specific monoclonal antibodies (Yi et al. 2013). A possible explanation for the observed discrepancy between mRNA and protein levels may be involvement of a yet undescribed mechanism regulating Lgr4 protein stability in the colon. In the stomach, 3HA–Lgr4 was visualized in the isthmic area of the glandular part directly under the gastric pits (Fig. 2a; E, E′, F, F′). We did not note any staining in the non-keratinized stratified epithelium of the nonglandular stomach reported previously by Barker and colleagues (Barker et al. 2010) or Mazerbourg and co-workers (Mazerbourg et al. 2004). The staining was based on the LacZ-reporter or immunohistochemistry, respectively. Of note, in the Barker’s article, the expression pattern of Lgr4 in the stomach was mentioned in the text only without any accompanying images. Finally, outside of the gastrointestinal tract we performed 3HA detection in the skin of Lgr4^3HA/3HA^ and control Lgr4^+/+^ mice. In the skin, Lgr4 has been described as a marker of hair stem cell expressed in the external root sheath (Kinzel et al. 2014; Mohri et al. 2008). As shown in Fig. 2c; G, G′, H, H′, the staining pattern of the 3HA tag in the skin of newborn mice reproduced well the published data.

Due to the fact that that TALENs-mediated cutting of genomic DNA starts in the dividing morula rather in the zygote (Li et al. 2014), the founder mice were genetic mosaics, i.e., more than two different alleles were detected in one mouse. Subsequent sequencing disclosed that the first founder in fact harbored four different alleles: wt, 3HA-modified, and two alleles with deletion 118 (including the first methionine deletion) and 34 nucleotides in the *Lgr4* genomic sequence adjacent to the site targeted by TALENs. Similarly, the second founder harbored four alleles: wt, 3HA-modifed, 117 bp (including the first methionine deletion) and 29 bp deletions. The third founder carried three alleles: wt, 3HA-modified, and 38 bp deletion generating a frame-shift mutation causing premature translation stop after codon 64. Since all three founders were crossed with wt mice next to Lgr4^3HA/3HA^ mice, Lgr4 null animals were generated. The Lgr4-deficient strains were named according to the size of the Lgr4 deletions as Lgr4^∆38/∆38^, Lgr4^∆117/∆117^, and Lgr4^∆118/∆118^ mice (Fig. 2b). In all three cases, the knockout allele was transmitted to the next generation, but mating of heterozygotes did not result in production of Lgr4^−/−^ knockout mice. We screened 12 l (64 animals in total) by PCR genotyping (primers P5 and P6) and obtained 20 (31%) wt, 44 (69%) heterozygotes, but no Lgr4-deficient animal. It was noted previously that the phenotype of an Lgr4 null animal is pleiotropic, dependent on the genetic background (Mazerbourg et al. 2004; Mendive et al. 2006). In C57BL/6 J mice, the Lgr4 absence (we employed Lgr4^∆38/∆38^ and Lgr4^∆118/∆118^ mice for the analysis) was manifested by various defects starting from embryonic day 13.5 (E13.5). At E18.5, the abnormalities included disturbed gut tissue, smaller bones with no evidence of mineralization, and the lungs were poorly developed with no signs of air sacs (not shown). Figure 2c shows typical pathological changes observed in the skin manifested by thin epidermis, substantially reduced number of hair follicles and lower number of proliferating cell nuclear antigen (PCNA)-positive cells.

### Cell biology and biochemical assays using Lgr4^3HA/3HA^ mice

**Fig. 3:**
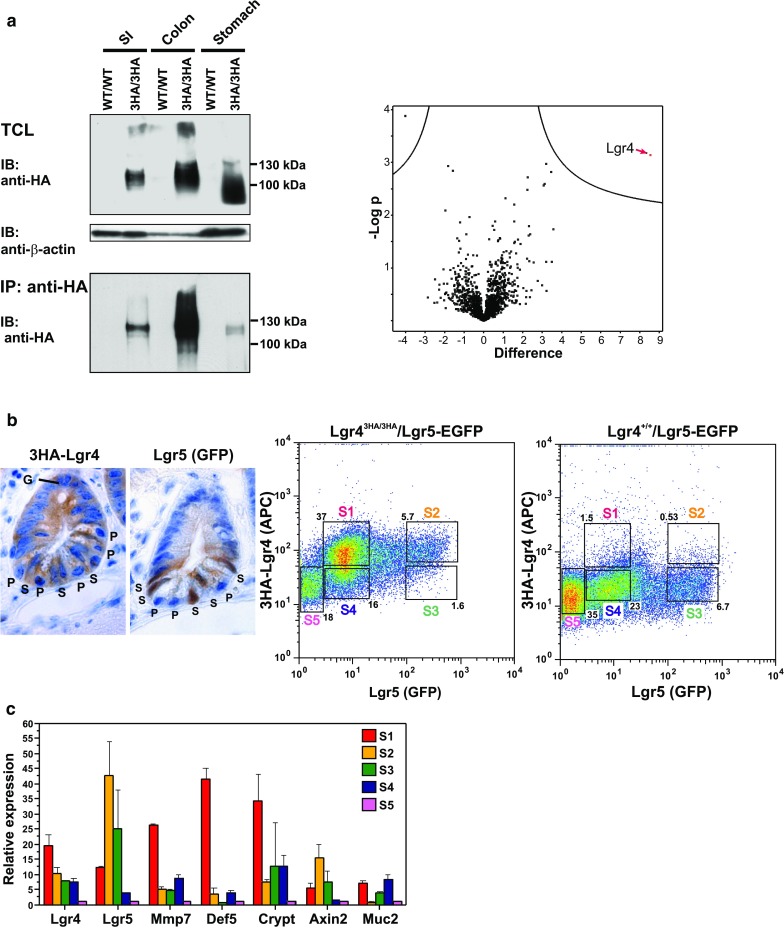
Biochemical analysis and cell sorting using Lgr4^3HA/3HA^ mice. **a** *Left* 3HA–Lgr4 protein detected by immunoblotting (*IB*) and immunoprecipitation (*IP*) using an anti-HA antibody. Total cell lysate (*TCL*) from the small intestinal (*SI*) and colonic crypts, and entire stomach were employed; anti-β-actin, loading control. *Right* MS from IP samples (small intestine) represented by the Volcano plot. *Y axis* negative log10 *p* value from *t* test; *X axis* log2 fold change between samples obtained from wt mice (WT/WT) or Lgr4^3HA/3HA^ (3HA/3HA) mice. Three IPs from wt and Lgr4^3HA/3HA^ samples were analyzed. Lgr4 was the only protein significantly enriched in 3HA/3HA IP (*red arrow*). Identified peptide counts 17; MS/MS 42; difference of label-free quantification (LFQ) intensity medians of samples derived from Lgr4^3HA/3HA^ and wt mice in binary algorithm 7.19299; fold change 146.3206927. **b** *Left* immunohistochemical detection of Lgr4- and Lgr5-positive cells in the lower portion of the small intestinal crypt obtained from an Lgr4^3HA/3HA^/Lgr5–EGFP mouse. Whereas Lgr5-positive cells are putative stem cells (*S*), Lgr4 is expressed in the majority of crypt cells including Paneth cells (*P*). The specimen also contains a vacuolized mucus-producing goblet cell or its precursor (*G*). *Right* representative experiment from fluorescence-activated cell sorting (*FACS*). Crypt cells from the small intestine of an Lgr4^3HA/3HA^/Lgr5–EGFP (*left panel*) or Lgr4^+/+^/Lgr5–EGFP mouse (*right panel*) were stained with a biotinylated anti-HA antibody, incubated with streptavidin APC-conjugated antibody and analyzed by FACS. Five cell populations (S1, S2, S3, S4, and S5) were gated in the samples. **c** Expression profiling of total RNA isolated from the cell population as indicated. Results were normalized to *ubiquitin B* expression. Relative abundance of gene expression in the indicated cell population versus the corresponding transcript level in cells present in the S5 population is indicated. Representative results of one experiment, which was repeated three times, are shown. *Error bars* indicate standard deviation. *Crypt* cryptidins 1,3,6–12,14,15, *Def5* defensin alpha 5, *Mmp7* matrix metallopeptidase 7, *Muc2* mucin 2. (Color figure online)

Our intention was to employ the tagged Lgr4 variant in various cell biology and biochemical assays. In this part we will provide several examples of such experiments. Isolated epithelial cells from the stomach, small intestine, and colon of wt and Lgr4^3HA/3HA^ mice were used to prepare total cell lysates (TCL) and perform IB and IP using an anti-HA tag-specific antibody. As shown in Fig. 3a, the procedures revealed a broad band corresponding to a protein of a putative size between 100 and 130 kDa in TCLs derived from the small intestine and colon of Lgr4^3HA/3HA^ but not wt mice. The size of the specific signal in TCL from the stomach was somewhat broader and smaller, probably due to partial degradation of the sample. A (relatively) weaker and sharp band matching the size of the protein present in TCLs from the small intestine and colon was obtained upon IP from the stomach sample, indicating N-terminal degradation of 3HA–Lgr4 in the lysate. A portion of the material retained on the anti-HA antibody-conjugated beads was also digested by trypsin and subjected to MS. In this pilot assay we used a limited amount of the material, and thus the only polypeptide detected (specifically) in the sample from Lgr4^3HA/3HA^ small intestine was Lgr4. Nevertheless, the assay confirmed the feasibility of the technique for future large-scale experiments (Fig. 3a).

Since the 3HA tag is expressed on the cell surface, we crossed Lgr4^3HA/3HA^ mice with Lgr5–EGFP–IRES–CreERT2 animals containing the EGFP–IRES–CreERT2 expression cassette inserted into the *Lgr5* locus (the strain is designated as Lgr5–EGFP). Consequently, EGFP production can be used as a surrogate stem cell marker (Barker et al. 2007). Cells isolated from the small intestinal crypts were labeled with an anti-3HA tag-specific antibody and visualized by red fluorescent dye APC-conjugated secondary antibodies, and subjected to cell sorting according to red (APC) and green (GFP) fluorescent signal intensity. The sorted cells were divided into five “gates” and collected. While 3HA–Lgr4 single-positive cells (population S1) constituted 37% of all sorted cells in Lgr4^3HA/3HA^/Lgr5–EGFP mice, the corresponding population in Lgr4^+/+^/Lgr5–EGFP constituted a negligible number (1.5%) of cells. Similarly, the double-positive cell Lgr4^+^/Lgr5^+^ population (S2 gate) constituted 5.7% cells in Lgr4^3HA/3HA^/Lgr5–EGFP crypts, but only 0.53% cells in Lgr4^+/+^/Lgr5–EGFP samples, indicating the specificity of the 3HA tag-specific surface labeling. In contrast, Lgr5^+^ single-positive cells (S3) representing 6.7% in Lgr4^+/+^/Lgr5–EGFP constituted a minor fraction (1.8%) in Lgr4^3HA/3HA^/Lgr5–EGFP. This shows that Lgr5 single-positive cells are rare and in most cases, Lgr5^+^ cells co-express Lgr4. Finally, two gated populations designated S4 and S5 represented Lgr4/5 double-negative cells of bigger or smaller size, respectively. The broader expression pattern of Lgr4 in the crypt bottom, in comparison, was also confirmed by immunohistochemical detection (Fig. 3b). Next, we isolated total RNA from the S1–S5 sorted cell populations obtained from Lgr4^3HA/3HA^/Lgr5–EGFP crypt and performed qRT-PCR analysis using several epithelial cell lineage-specific markers (Fig. 3c). Since S5 cells express neither of the markers, we concluded that they represent (differentiated) enterocytes. As expected, single-positive Lgr4^+^ cells (S1 population), next to high levels of *Lgr4* mRNA, also produced Paneth cell markers such as *matrix metallopeptidase 7* (*Mmp7*) and *alpha*-*defensin 5* (*Def5*) and multiple-*alpha defensins* detected by a common primer pair (designated as *Crypt*) (VanDussen and Samuelson 2010). Moreover, *mucin 2* (*Muc2*) was also enriched in the S1 population, indicating that it also contains goblet cell precursors (VanDussen and Samuelson 2010). The S2 Lgr4/5^+^ cells possibly include genuine intestinal stem cells (co)expressing (next to *Lgr4/5*) the Wnt target gene *Axin2*. The S3 population displayed low *Lgr4* expression and somewhat reduced levels of *Lgr5* and *Axin2*. Since these cells produced *Crypt*, they might represent the secretory precursors localized above the stem cell zone (Buczacki et al. 2013). Finally, the Muc2-positive goblet cells might be included in the S4 Lgr4/5 double-negative cell population. The population also contains cells positive for *Mmp7* and *Crypt*. However, the identity of the cells in unclear. We anticipate that the crypt cell lineage sorting will need (besides Lgr4 and Lgr5) more lineage-specific markers; nevertheless, surface Lgr4-specific labeling represents a good tool in this type of studies.

In summary, we generated a functional epitope-tagged *Lgr4* allele that can be used to identify Lgr4 interacting partners and/or visualize Lgr4-positive cells in various mouse organs and tissues.

## Electronic supplementary material

Below is the link to the electronic supplementary material.

Supplementary material 1 (PDF 71 kb)

## Abbreviations

3HA: Triple hemagglutinin
APC: Allophycocyanine
BMP: Bone morphogenic protein
Crypt: Multiple alpha defensins
DAB: 3,3′-diaminobenzidine
Def5: Alpha-defensin 5
IB: Immunoblotting
IP: Immunoprecipitation
LacZ: β-galactosidase
LGR: Leucine-rich repeat-containing G-protein-coupled receptor
Mmp7: Matrix metallopeptidase 7
Muc2: Mucin 2
MS: Mass spectrometry
PCNA: Proliferating cell nuclear antigen
PBS: Phosphate-buffered saline
qRT-PCR: Quantitative RT-PCR
RSPO: R-spondin
RT: Room temperature
TALEN: Transcription activator-like effector nuclease
TCL: Total cell lysates
Wt: Wild-type
