# Supplementary material for: Establishment of a tagged variant of Lgr4 receptor suitable for functional and expression studies in the mouse
Source: Transgenic Res. 2017 Jun 20;26(5):689–701. doi: 10.1007/s11248-017-0027-0 (PMC5602029; doi:10.1007/s11248-017-0027-0)
Supplement: Supplementary file 1 — Supplementary material 1 (PDF 71 kb) [file 11248_2017_27_MOESM1_ESM.pdf]

Supplementary Table S1

Usage	Oligo name	Sequence	Product size (bp) WT/3HA
Primer evaluating correct targeting	P1	TCTGTCTACCTTCAATACACAACCTGACAT	1170/1263
	P2	AAAAAGCTCCTAGAGGTTCAGATTCAAAGT	
	P3	TAGTTTTGCATGTACCTAAATGATTTGCAT	1462/1555
	P4	AGAACTTTCTCTCCCAACATTCTCTACCTC	
Primers for regular genotyping	P5	GGAGGCGAGTCGAGCGAGAGGAG	345/252
	P6	GCACTCACAGTGCTTGGGTGAAGGC	
qRT-PCR	Lgr4	AACCTGGAAACCCTGGACTT	124
		CTCCATCCGGGATAACAGAA	
	Lgr5	CCTGTCCAGGCTTTCAGAAG	173
		CTGTGGAGTCCATCAAAGCA	
	Mmp7	GGCCTAGGCGGAGATGCTCACT	77
		AACAGGAAGTTCACTCCTGCGTCC	
	Def5	TTCTCCAGGTGACCCCCAGCC	179
		GCAGACCCTTCTTGGCCTCCAAAG	
	Crypt	AGGAGCAGCCAGGAGAAG	192
		ATGTTCAGCGACAGCAGAG	
	Axin2	TAGGCGGAATGAAGATGGAC	103
		CTGGTCACCCAACAAGGAGT	
	Muc2	GGCCTCACCACCAAGCGTCC	118
		CGAAGGCGTGGCACTGGGAG	
	Ubiquitin B	ATGTGAAGGCCAAGATCCAG	160
		TAATAGCCACCCCTCAGACG	
